# Burden of herpes zoster: the direct and comorbidity costs of herpes zoster events in hospitalized patients over 50 years in France

**DOI:** 10.1186/s12879-015-1059-9

**Published:** 2015-08-19

**Authors:** Cecile Blein, Gaetan Gavazzi, Marc Paccalin, Charles Baptiste, Gilles Berrut, Alexandre Vainchtock

**Affiliations:** HEVA, Lyon, France; Université de Grenoble-Alpes et GREPI, clinique universitaire de médecine gériatrique, CHU de Grenoble, la tronche, France; Geriatrics Department University Hospital La Milétrie, Poitiers, France; Sanofi Pasteur MSD, Lyon, France; Hôpital Saint Jacques, CHU de Nantes, Nantes, France

**Keywords:** Herpes Zoster, Direct cost, Hospitalization, French, Indirect cost

## Abstract

**Background:**

The objectives of this study were to describe hospital stays related to HZ and to evaluate the direct and indirect cost of hospitalizations due to HZ among patients aged over 50 years.

**Methods:**

The hospitalizations of people aged over 50 years were selected from the French national hospital 2011 database (PMSI) using ICD-10 diagnosis codes for HZ.

Firstly, stays with HZ as principal or related diagnostic were described through the patient characteristics, type of hospitalization and the related costs. Secondly, a retrospective case–control analysis was performed on stays with HZ as comorbidity in 5 main hospitalizations causes (circulatory, respiratory, osteo-articular, digestive systems and diabetes) to assess the impact of HZ as co-morbidity on the length of stay, mortality rate and costs.

**Results:**

In the first analysis, 2,571 hospital stays were collected (60 % of women, mean age: 76.3 years and mean LOS: 9.5 days). The total health assurance costs were 10,8 M€. Mean cost per hospital stay was 4,206€. In the second analysis, a significant difference in LOS and costs was shown when HZ was associated as comorbidity in other hospitalization’s causes.

**Conclusions:**

HZ directly impacts on the hospital cost. When present as comorbidity for other medical reasons, HZ significantly increases the length of hospital stay with subsequent economic burden for the French Health System.

## Background

Herpes zoster (HZ), also called “shingles,” results from reactivation of latent varicella-zoster virus (VZV), after a primo-infection known as “varicella” or “chickenpox”. It spreads from a single ganglion to the neural tissue of the affected segment and the corresponding cutaneous dermatome [1].

If the reactivation is not contained, as can occur with age-related immunosenescence or iatrogenic immunosuppression, then viral replication ensues, resulting in ganglionitis, extensive infection, destruction of neurons and supporting cells [2]. This significant infection and associated inflammatory response is probably the origin of the prodromal pain that precedes the characteristic dermatomal eruption of HZ. Approximately 70 %–80 % of patients with HZ describe prodromal pain in the dermatome where skin lesions subsequently appear. Prodromal pain may be constant or intermittent and frequent or sporadic, and it may or may not interfere with sleep. The rash associated with HZ has a brief erythematous and macular phase, which is often missed, after which papules rapidly appear. These papules develop into vesicles within 1–2 days, and vesicles continue to appear for 3–4 days [1].

Increasing age is the primary risk factor for herpes zoster. The disease usually occurs after 50 years of age, and approximately 60 percent of cases occur in women [3, 4].Other risk factors include human immunodeficiency virus infection, neoplasic diseases, organ transplantation, use of immunosuppressive drugs, and other conditions that cause a decline in cell-mediated immunity. Complications occur in almost 50 % of older persons (60 years of age or older) with HZ [5–7]. Most frequent complications are persistent and neurological pain, named post-herpetic neuralgia (PHN), which concern 18 % of adults with herpes zoster and 33 % of those aged 79 and olders [8].

Herpes zoster ophthalmicus designates a localisation of herpes zoster with eye's involvement. Eyes complications are common in this case and result in considerable health care use and permanent vision decrement in about 6.6 % of individual with HZ eyes involvement [9].

Throughout Europe, estimated annual HZ incidences range from 2.0 to 4.6/1,000 person-years (PY) with lower values in Iceland, Germany and Switzerland (around 2/1,000 PY) and higher in Belgium, Spain, Italy (around 4/1,000 PY) and globally concerning women. The incidence and severity of HZ increase with advancing age; more than half of all persons in whom HZ develops are older than 60 years [10–14]. HZ epidemiological data are annually estimated by the French sentinel surveillance reporting system (based on data derived from consultations of a sample of French GPs). Moreover, HZ can be a repetitive history: in contrast with previous assessments, rates of HZ recurrence appear to be comparable to rates of first occurrence in immunocompetent individuals [15].

In France, estimated HZ annual incidences were 346,988 cases in 2009 [16], 269,833 cases in 2010 [17], 219,823 cases in 2011 [18] and 303,625 cases in 2012 [19]. Based on the sentinel surveillance reporting system, Gonzalez Chiappe *et al*., estimated the French 2010 HZ incidence rate to be 382 cases/100,000 inhabitants. The article also studies herpes zoster hospitalizations trough the PMSI database between 2000 and 2006. Over the seven years, 61,429 hospital stays were collected with a mean annual of 8,728 stays per year. The mean age observed was 72 years and mean LOS was 9.18 days [20].

The objectives of this study were to describe hospital stays related to HZ in 2011, and to evaluate the cost of hospitalizations due to HZ and the impact of zona as comorbidity on length of stay, death and cost in hospitalization for others diseases among patients aged over 50 years.

## Methods

### Data sources

The French Medical Information System (Programme de Médicalisation des Systèmes d'Information – PMSI) is an exhaustive medico-administrative hospital discharge database that covers all public and private hospitals in France as well as those in the French Territories [21–26]. Diagnoses identified during admission are coded using the International Classification of Diseases, 10th revision (ICD-10) by the physician. PMSI includes a compilation of standard discharge summaries (“Résumé Standard de Sortie”, RSS) for every admission. Anonymised data (“Résumé Standardisé Anonymisé”, RSA) with limited socio-demographic information (gender, age, residence code) and medical information on the main diagnosis that led to hospital admission, the nature of treatments received and examinations carried out, underlying comorbidities and possible complications, are made available for epidemiologic studies. Each patient's stay is classified by Diagnosis Related Group (DRG) (Groupe Homogène de Séjours) according to the information documented by the physician.

The economic burden of hospitalized cases would be expected to be well documented within the PMSI database because since the introduction of a DRG-based prospective payment system (the “Tarification à l’Activité”) in 2005, the PMSI database has been used as the basis for the funding of services in all hospitals, with each hospital receiving DRG-based payments according to the national tariff. Thus, data extracted from this database is exhaustive (all public and private hospitals are included and no sampling is done) and of high quality, with limited coding errors.

### Data collection of herpes zoster

All hospital stays with a primary, related and associated herpes zoster specific code were selected from the 2011 PMSI database using the ICD-10 codes B02* (“Zoster)”. In order to evaluate the hospitalization economic impact of HZ among patients aged over 50 years in France and to assess the impact on death, hospitalization’s length of stay and hospitalization’s cost of HZ when this latter is comorbidity in other hospitalization’s causes, two different data collection were done from the PMSI database.

For the first analysis, a conservative approach was done by selecting only those hospital stays that had a primary diagnosis or had direct link with HZ for patients aged over 50 years. HZ ICD 10 codes were selected based on Gonzalez Chiappe *et al*. in 2010 publication, by seven codes: B020 “HZ encephalitis”, B021 ”HZ meningitis”, B022 ”HZ with neurological disease”, B023 ”HZ Ophthalmicus”, B027 ”Disseminated HZ”, B028 ”HZ with other complications” and B029 ”HZ with no complications” [20]. A physician manually assessed all hospital stay cases that have an associated link to HZ (i.e. where HZ was a secondary diagnosis) to exclude those where HZ was considered doubtful.

For patient’s hospitalized for HZ, gender, age and comorbidities, type of management received (medical, surgical or exploratory), type of stay (conventional inpatient stays or short outpatient stays), and length of stay, type of HZ, immunosuppression factors, hospital discharges and occurrence of related deaths were collected. Conventional inpatient stays include day hospitalizations defined as an admission of 2 or more days’ duration, whereas short stays include day hospitalizations. Since patients may have several hospital stays during the year, the overall number of patients hospitalized at least once over a given period could be obtained by linking all hospital stays with anonymized patient identification numbers based on the patient’s social security number, date of birth and gender.

For the second analysis, all hospital stays with an associated HZ diagnosis code were firstly selected. Then immunocompromised patients have been excluded. Codes of immunosuppressive conditions were D8* “Certain disorders involving the immune mechanism“; B20* ”Human immunodeficiency virus [HIV] disease resulting in infectious and parasitic diseases“ to B24* “Unspecified human immunodeficiency virus [HIV] disease”. A disease approach was done by selecting only all the hospital stays in 5 main diseases where HZ is only a comorbidity diagnosis and not the reason for hospitalization. The first five reasons of hospitalizations observed with a HZ comorbidity were hospitalizations for circulatory system (14 %), hospitalizations for respiratory system (11 %), hospitalizations for osteo-articular system (6 %), hospitalizations for digestive systems (6 %) and hospitalizations for diabetes system (2 %).

For patients hospitalized for the 5 main diseases with HZ comorbidity, gender, age, were collected. A retrospective case–control was performed for each of the pre-selected 5 pathologies excluding hospital stays with at least an immunosuppression diagnosis. The cases were defined by hospitalizations with HZ in DAS and controls by cases-matched hospitalizations without HZ in DAS. Chi-square tests were done to compare the distribution of both genders and age between the two cohorts. A matching method based on age and sex was performed to neutralize confounding factors. Then median LOS, hospital mortality and costs were calculated for both selections and compared by statistical non-parametric analyses (Wilcoxon-Mann-Whitney) in each of the five categories.

### Economic burden of HZ hospitalization

Costs were estimated from the social security payer perspective, i.e. Ministry of Health public fund. Ambulatory costs and indirect costs related to productivity loss were not considered in the present study. Hospital-associated costs were calculated using official DRG tariffs for each year considered. DRG tariffs represent the willingness-to-pay by the national health insurance and not the hospital cost production. DRG tariffs include medical and related procedures, nursing care, treatments (except specific expensive drugs), food and accommodation, and investment costs for hospitalized patients. Additional cost per day of hospitalization in emergency or an intensive care unit was added to DRG tariffs, when appropriate. For private hospitals, physician’s fees were also added to the DRG tariffs; physicians are reimbursed on a fee-for-service basis [source: ENCC 2010]. Costs are presented as mean/median cost per stay, mean/median cost per patient and total cost per year for France. All costs are presented in actualized Euros (base year 2012).

### Ethics and consent

This non-interventional study does not fall under the scope of the law and does not require any ethics committee submission (Law 88–1138 relative to Biomedical Research of December 20, 1988, modified on August 9, 2004). The ATIH (*Agence Technique de l*’*Information sur l*’*Hospitalisation*) is responsible for managing the finalized database each year under approval by the CNIL (National commission for data processing and civil liberties).

## Results

### Burden of HZ hospitalization

#### Hospital stay characteristics

**Fig. 1:**
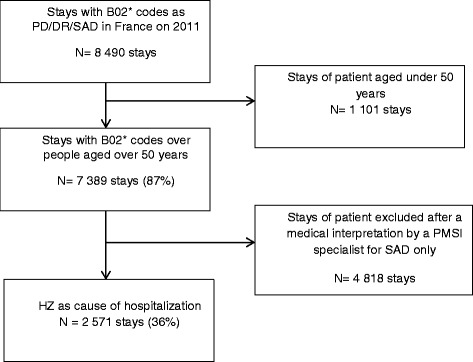
Chart flow of study protocol of HZ as cause of hospitalization

A total number of 7,389 stays for HZ were identified in the PMSI 2011 database for patients aged over 50 years. A medical interpretation by a DIM physician classified the selection into 2 groups, 2,571 hospital stays (35 %), related to 2,392 patients, were directly related to HZ (2,362 and 209 stays were in public and private hospitals, respectively), and 4,818 stays (65 %) were indirectly related to HZ. This last group was excluded (4,047 and 771 stays were in public and private hospitals, respectively) [Fig. 1]. A conservative approach was taken by selecting only those hospital stays that had a primary diagnosis of HZ or had direct link with HZ.

Over the 2,571 hospital stays observed during the study period for HZ, stays for B029 “HZ without complications” was mainly represented with 911 hospital stays (33.1 %). The following type of zona B023 “HZ Ophthalmicus”, B028 “HZ with other complications”, and B022 “HZ with neurological disease” represented between 450 and 487 stays (17.5 % to 18.9 %). Others type of zona represented between 147 and 44 hospital stays [Table 1].

**Table 1:** Hospital stays, LOS and means HZ Costs for Health Assurance (€)

	B020 “HZ encephalitis”	B021 “HZ meningitis”	B022 “HZ with neurological	B023 “HZ Ophtalmicus”	B027 “Disseminate d HZ”	B028 “HZ with other complications	B029 ”HZ with no complications	Total
Public hospital stays	59	42	407	463	136	417	838	2,362
Private hospital stays	4	2	43	24	11	52	73	209
Total stays	63	44	450	487	147	469	911	2,571
% stays	2.5	1.7	17.5	18.9	5.7	18.2	35.4	100
LOS	19.5	14.3	9.8	9.1	13.0	9.8	7.9	9.5
Mean Costs per stay in public hospital	8,912 €	5,122 €	4,597 €	3,587 €	7,269 €	4,848 €	3,568 €	4,350 €
SD	6,847 €	3,143 €	3,138 €	2,112 €	25,889 €	2,592 €	3,219 €	6,963 €
Median Costs per stay in public hospital	8,304 €	4,206 €	3,819 €	3,426 €	5,205 €	5,765 €	3,702 €	3,701 €
Range	38,302 €	15,461 €	20,375 €	13,334 €	302,233 €	21,434 €	77,402 €	302,645 €
Mean Costs per stay in private hospital	8,605 €	3,662 €	3,139 €	2,139 €	2,187 €	2,442 €	2,471 €	2,677 €
SD	4,698 €	230 €	1,880 €	1,194 €	1,248 €	1,324 €	1,785 €	1,897 €
Median Costs per stay in private hospital	6,455 €	3,662 €	2,414 €	2,367 €	2,519 €	2,519 €	3,219 €	2,519 €
Range	9,770 €	325 €	681 €	4,962 €	3,570 €	5,868 €	6,136 €	15,341 €
Mean Costs per stay	8,893 €	5,056 €	4,458 €	3,516 €	6,888 €	4,581 €	3,480 €	4,206 €
SD	6,703 €	3,084 €	3,069 €	2,098 €	24,932 €	2,595 €	3,142 €	6,711 €
Median Costs per stay	8,304 €	4,206 €	3,819 €	3,427 €	3,869 €	5,765 €	3,702 €	3,702 €
Range	38,032 €	15,461 €	20,375 €	13,334 €	302,484 €	21,434 €	77,402 €	302,646 €
Total Costs	560,284 €	222,465 €	2,006,179 €	1,697,544 €	1,007,870 €	2,148,890 €	3,167,679 €	10,810,911 €
Proportion of the total HZ cost	5 %	2 %	19 %	16 %	9 %	20 %	29 %	100 %

HZ: Herpes Zoster, LOS: Length of stay, SD: Standard Deviation

Sex ratios were equivalent in almost all HZ type. Women represented 60 % of patients of the cases. We observed a different sex ratio only in the B027 “Disseminated HZ” type where the women represented 50 % of patients.

Over the 2,392 patients included, the overall mean age was 76.3 years (SD 11.7) and the median age was 79 years (range 57) [Table 2]. The mean age was different between HZ types : 77.7 years (SD 11.1) for B028 “HZ with other complications”, 77.3 years (SD 11.2) for B023 “HZ Ophthalmicus”, 73.5 years (SD 12.1) for B027 “Disseminated HZ”, 73.3 years (SD 11.7) for B021 “HZ meningitis” and 74.2 years (SD 12.2) for B020 “HZ encephalitis”. Over the 2,392 patients, 444 patients (18.6 %) were aged 80–84 years, 438 patients (18.3 %) were aged 85–89 years, 339 patients (14.2 %) were aged 75–79 years, and 244 patients (10.2 %) were aged 70–74 years [Table 2]. Patients were mainly hospitalized once (95 %) during the study period of 1 year.

**Table 2:** Distribution of patients per age group and per HZ Code

Age group	B020 “HZ encephalitis”	B021 “HZ meningitis”	B022 “HZ with neurological disease”	B023 “HZ Ophtalmicus”	B027 “Disseminated HZ”	B028 “HZ with other complications”	B029 “HZ with no complications”	Total	%
50–54 years	6	2	19	18	11	14	44	113	4.7
55–59 years	4	7	32	22	16	24	69	171	7.1
60–64 years	3	3	30	38	13	34	83	201	8.4
65–69 years	8	5	34	30	14	30	81	193	8.1
70–74 years	6	0	48	45	19	50	78	244	10.2
75–79 years	9	7	64	75	17	73	105	339	14.2
80–84 years	11	8	84	91	23	88	146	444	18.6
85–89 years	12	8	75	84	25	83	161	438	18.3
90–94 years	3	1	30	45	6	44	67	190	7.9
95 years and over	0	0	6	12	2	13	29	61	2.5
Total (patients)	62	41	422	460	146	453	863	2,392	100
Mean age (years)	74.2	73.3	76.1	77.3	73.5	77.7	75.9	76.3	
SD	12.2	11.7	11.4	11.2	12.1	11.1	12.2	11.7	
Median age (years)	76,5	76	78	80	74	80	78	79	
Range	43	39	52	57	47	52	51	57	

HZ: Herpes Zoster, SD: Standard Deviation

**Fig. 2:**
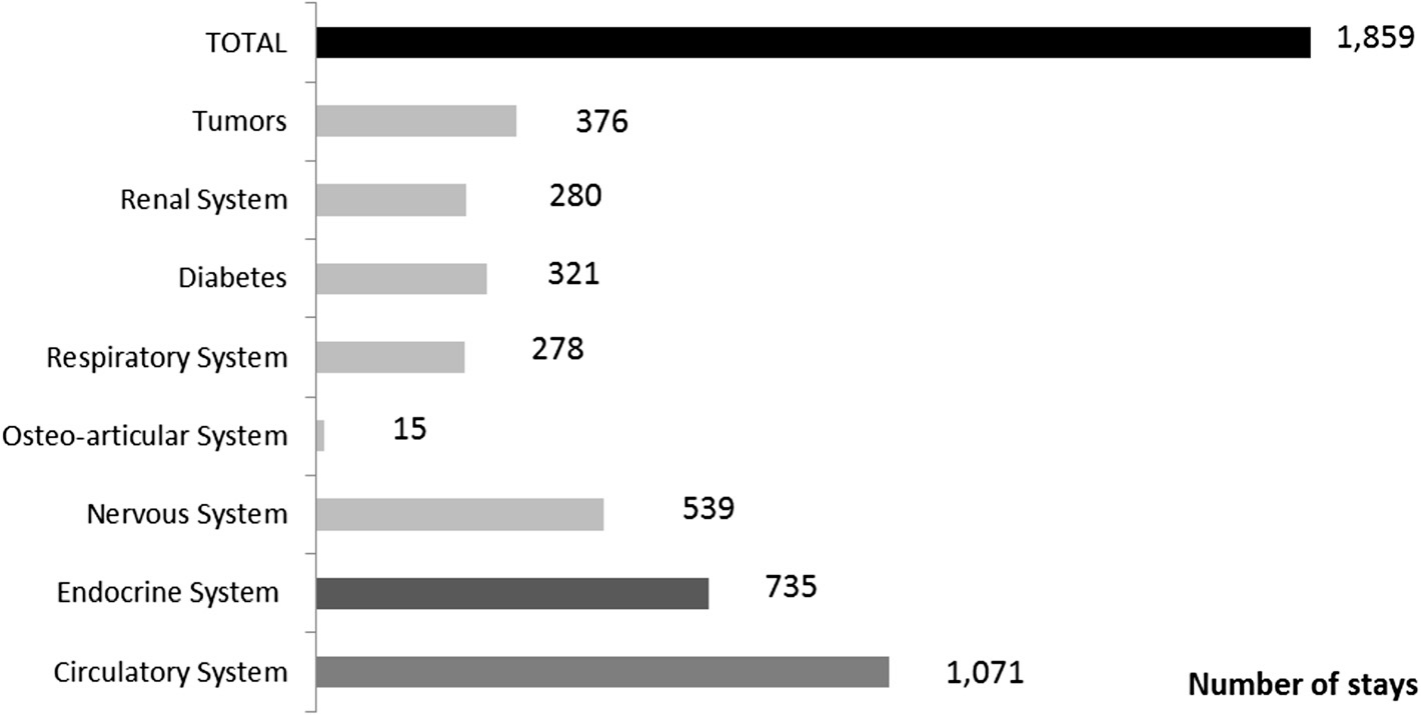
Distribution of comorbidity in Zona hospital stays

Public hospital represented 92 % (2,362) of hospital stays [Table 1] and in nearly all hospital stays (97 %; 2,250), the illness was medically managed, with surgical intervention recorded in <1 % (30) of stays. More than half of stays (72 %; 1,859) had comorbidity. Circulatory system was the main co-morbidity with 41.7 % (1,071), followed by endocrine system in 28.6 % (735), nervous system in 21.0 % (539) and malignancies in 14.6 % (376). [Fig. 2] Diabetes, kidney diseases and respiratory system co-morbidity rates ranged from 10.8 % (278) to 12.5 % (321) and finally musculoskeletal system occurred in 0.6 % (15). A patient may have several comorbidities within a stay, so the sum of different kinds of comorbidity stays does not give the total amount of stays. Of the 2,571 hospital stays, 81 (3.2 %) stays presented immunosuppression treatments codes, ranging from 0.0 % to 3.6 % except for the code B027

.

Conventional inpatient stays represented 44 % (2,408 stays) of all hospital stays. The mean length of hospital stays was 9.5 days (SD 11.22) and the median length of stay was 7 days (range from 0 to 385 days), ranging from 7.9 days (SD 7.46) for B029 “HZ without complications” to 19.5 days (SD 15.37) for B020 “HZ encephalitis” [Table 1].

A death rate of 1.6 % (38 patients) was observed during the study, range 0.5 % (5/911) for B029 “HZ without complications” to 7.9 % (5/63) for B020 “HZ encephalitis” [Table 3]. Over the 38 deaths observed, only 1 death occurred among patients with no comorbidity although 37 deaths occurred among patients with at least one comorbidity.

**Table 3:** Distribution

	Home		Transfered								Unit mutation		Death		Total
			MCO		SSR		USLD		Total						
	Stay	%	Stay	%	Stay	%	Stay	%	Stay	%	Stay	%	Stay	%	
B020”HZ encephalitis”	31	49.2 %	9	40.9 %	12	54.5 %	1	4.5 %	22	34.9 %	5	7.9 %	5	7.9 %	63
B021 “HZ meningitis”	28	63.6 %	4	40.0 %	6	60.0 %	0	0.0 %	10	22.7 %	5	11.4 %	1	2.3 %	44
B022 “HZ with neurological disease”	356	79.1 %	14	29.8 %	31	66.0 %	2	4.3 %	47	10.4 %	39	8.7 %	8	1.8 %	450
B023 “HZ Ophtalmicus”	398	81.7 %	18	33.3 %	32	59.3 %	4	7.4 %	54	11.1 %	25	5.1 %	10	2.1 %	487
B027 “Disseminated HZ”	118	80.3 %	7	41.2 %	9	52.9 %	1	5.9 %	17	11.6 %	9	6.1 %	3	2.0 %	147
B028 “HZ with other complications”	372	79.3 %	18	35.3 %	32	62.7 %	1	2.0 %	51	10.9 %	40	8.5 %	6	1.3 %	469
B029 “HZ with no complications”	757	83.1 %	33	37.5 %	51	58.0 %	4	4.5 %	88	9.7 %	61	6.7 %	5	0.5 %	911
% Sub-Total				35.6 %		59.9 %		4.5 %		100 %					
Total	2,060	80.1 %	103	4.0 %	173	6.7 %	13	0.5 %	289	11.2 %	184	7.2 %	38	1.5 %	2,571

HZ: Herpes Zoster, LOS: Length of Stay, MCO: Medicine Surgery Obstetrics, SSR: After-care and rehabilitation, USLD: Long-Term Care Unit

Discharge from hospital to home occurred in 2,060/2,571 hospital stays (80.1 %) [Table 3]. Alternatively, 289 hospital stays (11.2 %) led to a transfer into another health care institution (173 stays in a Recovery after care and rehabilitation Center). Intra hospital transfer occurred in 184 hospital stays (7.2 %) and led to an internal transfer between units within the same hospital. The rate of discharge is very high for HZ except for the code B020 “HZ encephalitis” (31/63 stays or 49.2 %) and B021 “HZ meningitis” (28/44 stays or 63.6 %) The latters have a higher rate of transfer to another health care institution with respectively 34.9 % for B020 and 22.7 % for B021 [Table 3].

Over the 2,571 stays, 2,446 hospital admissions (95 %) from home patient were mainly observed. Among the 473 hospital discharges to transfer or mutation (289 transfers / 184 mutations), 419 hospital admissions (88 %) from home patient were collected.

### Economic burden of HZ hospitalization

Total HZ health insurance costs for the 2,571 hospital stays were estimated at responsible for 10,810,911€ [Table 1]. The mean costs for health insurance per hospital stay was 4,206€ (SD 6,211€) with a range from 4,350€ (SD 6,963€) for public hospital to 2,677€ (SD 1,897€) for private hospital. The mean cost per patient was 4,519€ (SD 6,905€) range to 4,653€ (SD 7,164€) for public hospital to 2,812€ (SD 1,888€) for private hospital.

The mean costs per hospital stay for HZ ranged from 8,893€ (SD 6,703€) for B020 “HZ encephalitis” to 3,480€ (SD 3,142€) for B029 “HZ without complications” [Table 1]. The total annual costs of B029 “HZ without complications” care were responsible for 29 % of total HZ annual costs, B028 “HZ with other complications” for 20 %, B022 “HZ with neurological disease” for 19 %, B023 “HZ Ophthalmicus” for 16 %, B027 “Disseminated HZ” for 9 %, B020 “HZ encephalitis” for 5 %, and B021 “HZ meningitis” for 2 % [Table 1].

### Impact of HZ comorbidity in hospitalizations for other reasons

#### Hospital stay characteristics

A total number of 4,818 hospital stays with a zona comorbidity among patients aged over 50 years without immunosuppression factors were identified in the 2011 PMSI database. Of the hospital stays with a zona comorbidity, only 1,717 hospital stays were selected corresponding to the 5 main reasons of hospitalizations : circulatory (ICD-10 codes: I0*-I5* & I7*-I9*), respiratory (J0*-J9*), osteoarticular (M0*-M9*), digestive systems (K0*-K9*), and diabetes (E1*). In order to evaluate the statistical impact of zona as comorbidity on length of stay, death rate, and cost, a separately case control was performed for each disease.

Chi-square tests showed that age and sex distribution were significantly different between the 2 cohorts for each disease (p < 0.001). As a consequence, extracted data were adjusted by age and sex for all disease [Table 4]. A case-cohort stay was matched with 3 randomly chosen control-cohort stays for the same age group, sex and pathology. The co-morbidity analysis was based on the selection of the 1.717 stays for patients aged over 50 years in cases cohort and of 5,151 stays out of the 3,665,088 stays of patients aged over 50 years in control cohort [Table 4].

**Table 4:** Data classification per hospitalization causes, ages, and sex

			Cases^a^		Total		Controls^b^		Total
		[50–75]	[75–85]	[85 years and over]		[50–75]	[75–85]	[85 years and over]	
Circulatory System	Men	81	89	62	232	243	267	186	696
	Women	57	133	193	383	171	399	579	1,149
	Total	138	222	255	615	414	666	765	1,845
Respiratory System	Men	86	89	73	248	258	267	219	744
	Women	59	89	85	233	177	267	255	699
	Total	145	178	158	481	435	534	474	1,443
Osteo-articular System	Men	44	30	15	89	132	90	45	267
	Women	62	51	66	179	186	153	198	537
	Total	106	81	81	268	318	243	243	804
Digestive System	Men	44	29	20	93	132	87	60	279
	Women	71	64	47	182	213	192	141	546
	Total	115	93	67	275	345	279	201	825
Diabetes	Men	23	13	2	38	69	39	6	114
	Women	8	20	12	40	24	60	36	120
	Total	31	33	14	78	93	99	42	234
Total		535	607	575	1,717	1,605	1,821	1,725	5,151

^a^Cohort of patients hospitalized with HZ as comorbidity

^b^Cohort of patients hospitalized without HZ comorbidity

#### HZ impact as co-morbidity in 5 main diseases

In each of the five diseases, i.e. circulatory, respiratory, osteoarticular system, digestive systems and diabetes, the median LOS presented a statistically significant difference between cases and control cohorts [Table 5]. For osteoarticular system, the median LOS differed by 3 days leading to a 50 % rise from control cohort to cases cohort. The median LOS differed by 4 days (+80 %) for diabetes and by 5 days (+71 %) for respiratory system. Then, the median LOS differed by 6 days for circulatory system (+120 %) and for digestive system (+300 %).

**Table 5:** HZ as co-morbidity analyses: LOS, death rate and economic evaluation

		Circulatory system	Respiratory system	Osteoarticulary system	Digestive system	Diabetes
LOS						
Median (days)	Cases^a^	11	12	9	8	9
	Control^b^	5	7	6	2	5
Median difference days		6	5	3	6	4
% rise		1.2 %	0.71 %	0.5 %	3 %	0.8 %
Tests (Wilcoxon-Mann–Whitney)	(p values 0,05)	<0.0100	<0.0001	<0.0001	<0.0001	<0.0001
Death rate						
Deaths among cases cohort^a^						
	Death rate (%)	32/615	41/481	5/268	16/275	1/78
Deaths among control cohort^b^		5.2 %	8.52 %	1.86 %	5.81 %	1.28 %
	Death rate (%)	113/1,845	155/1,443	2/804	26/825	3/234
Chi-square probability	(p value 0,05)	6.12 %	10.74 %	0.24 %	3.15 %	1.28 %
Hospital stay cost						
Median (€)	Cases cohort^a^	4,228	4,534	4,475	3,601	3,510
	Control cohort^b^	3,371	3,589	3,553	1,590	2,523
Median difference (€)		857	945	922	2,011	987
% rise		25 %	26 %	26 %	126 %	39 %
Tests (Wilcoxon-Mann–Whitney)	(p value 0,05)	<0.0100	<0.0001	<0.0001	<0.0001	<0.0001

HZ: Herpes Zoster

LOS: Length of stay

^a^Cohort of patients hospitalized with HZ in SAD

^b^Cohort of patients hospitalized without HZ in SAD

Statistically significant difference in median stay costs were demonstrated between cases and control cohorts [Table 5]. For circulatory system, median stay costs differed by 857€ (25 % rise in the case cohort compared to controls). Median stay costs differed by 922€ (+26 %), from 945€ (+26 %) and from 987€ (+39 %) for osteoarticular system, for respiratory system and for diabetes respectively. Then, the median hospital stay costs differed by 2,011€ for digestive system (+126 %).

No difference was observed in the death rates in 3 diseases: circulatory system, respiratory system and diabetes [Table 5]. Death rates for osteoarticular system varied for the 3 age groups: it was identical in both cohorts of [50-75] years groups; null in both cohorts of [75-85] years groups and significantly different (1.86 % for cases cohort and 0.24 % for control cohort) in the group of 85 years and over group.

## Discussion

The current PMSI 2011 analysis identified 2,571 stays of patients aged over 50 years hospitalized for HZ corresponding of 0.9 % of the 2011 recorded total stays, while diabetes represented 95,204 stays (62 times more) and degenerative disease represented 48,291 stays (19 times more). The HZ represents a subsequent economic burden of approximately 11 million€ in global Health Assurance expenses and quality management may reduce the total amount spent for HZ treatment. The total annual costs of B029 “HZ without complications” and B023 “Zoster Ocular Disease” care were responsible for 29 % and 16 % of total HZ annual costs respectively. The HZ stay mean cost is 4,206€ ranging between 3,480€ for HZ without complications to 8,893€ for Zoster encephalitis. The Zoster Ocular Disease stay mean cost of 3,516€ is similar to the cost observed in HZ without complications. The absence of cost impact between these two HZ is due to the classification DRG system. In the case of Zoster Ocular Disease stays, the DRG are mainly distributed in the Major Diagnostic Category (MDC) 02 therefore the HZ without complications stays are mainly distributed in the MDC 09. The DRG tariffs relevant to the MDC 02 are lower than the DRG tariffs of MDC 09.

Clinical and costs input data were derived from a cohort of 2,392 patients, with 60 % of women and with a mean age of 76.3 years. Death occurs in 1.6 % of cases of patients hospitalized with zoster. B020 “HZ encephalitis” and B021 “HZ meningitis” stays characteristics involved older patients, longer LOS, higher rates of transfer to another health care institution and higher death rates even though the related number of extracted stays was the lowest (63 and 44 stays respectively).

When present as only comorbidity in hospitalizations for other hospitalization causes, HZ significantly increases the LOS at hospital and subsequent economic burden for the French health system whereas death rates were not linear as expected and do not stand a classical conclusion. Comorbidity indicates the co-occurrence of preexisting age-related health conditions in reference to an index disease [27]. Advanced age is associated with increased vulnerability to chronic health problems. Given the complexity and heterogeneity involved in comorbidity, however, no single definition or measure would serve all research and clinical purposes. The level of comorbidity correlates with the magnitude of immune response in older adults (decrease in T cell proliferation and IL-12 production and increase in IL-10 production in response to PHA stimulation) [28]. Until recent published papers, comorbidities were not taken into account in the management and prognosis of herpes zoster [29]. Risk of herpes zoster has been found in patients with chronic obstructive pulmonary disease [30], kidney disease [31, 32], rheumatoid arthritis [33], inflammatory bowel disease [34], type II diabetes [35]. In addition, some authors report a risk of cancer after infection by herpes zoster [36, 37]. Few data analyzed impact of comorbidities on the management and the cost of herpes zoster.

A Spanish study has been conducted by Gil-Prieto *et al*. in 2011, describing the disease burden of patients aged ≥50 years hospitalized with HZ (in DP or DAS) between 1998 and 2004 who presented Chronic Obstructive Pulmonary Disease (COPD), cardiovascular disease and/or diabetes [38]. The annual number of hospitalization was 2,289 stays with a mean LOS of 12.4 days. Death rates were 3.7 % and increased to 4.7 % for patients with comorbidity even though death rates were 2.1 % for the cohort without comorbidities. Death rates were higher due to a higher death probability in the treatment of COPD, cardiovascular disease and/or diabetes as the cohort involved patients hospitalized for another primary diagnosis (COPD, cardiovascular disease and/or diabetes).

The study shows only than more women than men with HZ are hospitalized and hospitalization is prevalent in older ages, which is in line with epidemiological studies. As reported by Gonzalez Chiappe *et al*. [20] and by Chang *et al*. two decades ago for an Asian population [39], the predominance of HZ in women might be ascribed to a higher proportion of females in the older population. Moreover, Thomas *et al*. added, in 2004, that women might be more likely to seek medical advice, thereby causing higher reporting rate [32]. Pinchinat *et al*. review stated, in 2013, that HZ incidence increases sharply with age for the European population [12, 40].

The present study method included a check step by analyzing the age and sex characteristics and their statistical differences in both groups with HZ and without HZ in each of the five pathologies. This check step conclusion determined the need of matching for further analyses to neutralize the confounding factors.

This study highlighted the impact of an associated diagnosis of HZ for patients aged 50 years and older with chronic conditions. When associated to one of the 5 most common chronic diseases, HZ significantly increased the LOS. Herpes Zoster significantly increases length of hospitalization (p < 0.001) leading to subsequent economic burden for the French health system. It is expected that this extension of LOS also has an impact on the cost of the management of the disease. Herpes Zoster doesn’t significantly increase the number of deaths.

This review has various limitations. First of all, PMSI database is the basis of hospital funding and not an epidemiological register. The prospective payment is directly linked to the coding process leading to optimize this latter. Nevertheless the high exhaustiveness and quality of information (including some medical information) allows using the PMSI database to estimate various indicators by disease, like the number of patients hospitalized annually, the number of stays per patient and the total cost of hospitalization [24–26]. Using hospitals and death records generate limitations: codes were not necessarily accurate; i.e., misclassification or diagnosis errors by coding as a different herpes [41], under reporting of pre-existing conditions such as stroke [42]; inadvertent omissions, the unavailability of medical records to certifying physician and difficulties in determining the underlying cause of death and hospitalization when several disease processes were involved [43, 20].

Further investigations to study the impact of HZ comorbidty in other categories, to provide up-to-date analyses (PMSI 2013 follow-up) in the following 5 to 10 years, will allow a long-term projection of clinical characteristics, economic evaluation and trend over time.

## Conclusion

The present study evaluated the economic burden of HZ as the cause of hospitalization and as comorbidity. The total annual cost of HZ as cause of hospitalization was approximately 11 millions € and the mean costs per stay was 4,206€ (SD 6,711€). About a third (29 %) of the annual cost was explained by the B029 “HZ with no complications”.

When HZ was present as only comorbidity in five hospitalizations reasons (circulatory system, respiratory system, osteoarticular system, digestive system and diabetes), the hospitalizations length of stay and cost were significantly increased (p < 0.001). Median stay costs rose from 25 % to 126 % and median LOS from 3 to 6 days.

In a context of ageing population, where incidence of HZ increases dramatically after 50 years of age, HZ impact length of stay and contributes to demonstrating the clinical and economic impact of HZ. The hospitalization economic burden is only a part of the total cost of management of HZ.

## Abbreviations

ATIH: Agence Technique de l’Information sur l’Hospitalisation
CCAM: French Classification Of Medical Procedures or *Classification Commune des Actes Médicaux*
CNIL: National commission for data processing and civil liberties
COPD: Chronic Obstructive Pulmonary Disease
DAS: Secondary Associated Diagnosis or *Diagnostic Associé Significatif*
DIM: Department Of Medical Data or *Département d*’*Information Médicale*
DR: Associated Diagnosis or *Diagnostic Relié*
ENCC: National study of costs or *Echelle Nationale des Coûts à Méthodologie Commune*
HZ: Herpes Zoster
ICD-10: X^th^ Revision of the International Classification of Diseases
INPES: French Institute for Health Education and Prevention
INSEE: French Institute For Statistics And Economic Studies
LOS: Length of Stay
PMSI-MCO: French Computerised Hospital Database For Medicine, Surgery And Obstetrics or *Programme de Médicalisation des Systèmes d*’*Information*-*Médecine Chirurgie Obstétrique*
MDC: Major Diagnostic Category
PD: Principal Diagnosis or Diagnostic Principal
PY: person-years
RSA: Discharge Summary or *Résumé de Sortie Anonyme*
SSR: Recovery Center or *Soin de Suite et de Réadaptation*
T2A: Prospective Payment or *Tarification à l*’*Activité*
USLD: Long-Term Unit Care or *Unité de Soins de Longue Durée*
VZV: Varicella-Zoster Virus
